# Robust detection framework for adversarial threats in Autonomous Vehicle Platooning

**DOI:** 10.3389/fdata.2025.1617978

**Published:** 2025-11-19

**Authors:** Stephanie Ness

**Affiliations:** University of Vienna, Diplomatic Academy of Vienna, Vienna, Austria

**Keywords:** Autonomous Vehicle Platooning (AVP), active learning, machine learning classifiers, adversarial threat detection, anomaly detection

## Abstract

**Introduction:**

The study addresses adversarial threats in Autonomous Vehicle Platooning (AVP) using machine learning.

**Methods:**

A novel method integrating active learning with RF, GB, XGB, KNN, LR, and AdaBoost classifiers was developed.

**Results:**

Random Forest with active learning yielded the highest accuracy of 83.91%.

**Discussion:**

The proposed framework significantly reduces labeling efforts and improves threat detection, enhancing AVP system security.

## 1 Introduction

Self-organizing Intelligent Car Train (SICT) or Autonomous Vehicle Platooning (AVP) is a relatively new concept in which a group of self-driving cars follow each other at short intervals to improve fuel consumption, safety, and traffic conditions. The use of technology in which a vehicle communicates with another vehicle (V2V) and with infrastructure systems (V2I) about its speed, position, and desired routes (Gupta et al., 2020). However, the synergy and continuous integration in AVP indicate that it is also vulnerable to adversarial threats, which are deliberate attempts to compromise the confidentiality, integrity, availability, or security of the providing system (Abramson, 2016). Adversarial attacks are able to alter sensor inputs, signals or decisions and can cause the vehicle to perform uncontrollably and dangerously. This can lead to accidents or traffic jams (Gupta et al., 2020). Identifying these adversarial threats is especially important for the protection of self-driving car formations when deployed on the road. Therefore, the enhancement of a morphological approach to threat detection is crucial at this stage to prevent and counter related risks and guarantee stable functioning of the system (Sun N. et al., 2023). This paper demonstrates that adversarial attacks on Autonomous Vehicle Platooning (AVP) pose significant risks, compromising both safety and efficiency (Anwar, 2023). Because AVP relies on accurate sensor measurements and proper message exchange between its vehicles, an attack could tamper with this information, resulting in disastrous consequences (Abrar, 2023). For instance, a compromised platoon may suddenly change from accelerating to braking, resulting in multiple car pileups, jams or worse, fatal accidents. Predictably, those same adversaries can also break apart the platoon as it achieves an appropriate distance between vehicles to travel efficiently and reduce fuel consumption too, thus defeating the very purpose of environmental improvement (Loukas et al., 2019). Also, such attacks may shift from the preferred course of travel and make vehicles prone to hijackings or other malicious exploitation. In summary, the adversarial threats pose danger to the lives of the vehicles' occupants and other persons on the roads besides undermining public confidence in self-driving systems, thus hampering the fast expansion of such technology (Mehta et al., 2023). The importance of autonomous transportation systems' safety and performance motivates the detection of adversarial attacks on Autonomous Vehicle Platooning (AVP) (Soltaninejad and Rashidfarokhi, 2023). AVP is intended to improve road safety, traffic conditions, and fuel efficiency by allowing AVs to travel in a dense convoy. However, adversarial attacks pose a threat to these advantages because they can exploit flaws in communication and control systems to cause vehicles to behave erratically (Ahmad et al., 2024). This unpredictability can lead to scenarios such as accidents, traffic jams, and, in the worst-case scenario, the loss of life. Furthermore, adversarial attacks undermine trust and confidence, which are essential for the shift to autonomous systems. As a result, identifying these attacks is critical not only to protect passengers and surrounding traffic, but also to maintain the reliability of AVP systems (Ni et al., 2019). Now, maintaining or preventing such an attack ensures that the use of autonomous vehicle platooning is truly realized in revolutionizing current transportation systems as intended, without being subjected to such interferences (Chowdhury et al., 2020). Indeed, the implementation of sound detection capabilities serves not only the purpose of protection, but also the suitability of applications for the adaptability, expansion, and appeasement of Level 5 autopilot systems.

Active learning is a machine learning approach that selectively queries the labels of the most questionable data points and offers significant advantages for strategically countering hostile threats. Especially in frequently changing threat environments such as AVP scenarios, collecting tagged data can be very time-consuming and costly. Active learning, especially uncertainty sampling, reduces the annotation effort in a targeted manner by focusing labeling on the most informative examples. This method not only reduces the effort required for labeling, but also makes classifiers more flexible and accurate in detecting attacks. Recent reviews, such as Ren et al. (2021), Margraf et al. (2024), and Mienye and Sun (2022), emphasize the effectiveness of active learning techniques in real-time security applications with limited resources.

The contribution of this project is as follows:

- The paper presents a new concept that integrates active learning with multiple ML classifiers (Random Forest, Gradient Boosting, XG-Boost, KNN, logistic regression, AdaBoost). This integration enhances the performance of the method to detect adversarial threats in the Autonomous Vehicle Platooning (AVP) by a large measure.
- Specifically, our proposed method reduces the required amount of the labeled data and targets the most informative or uncertain data samples using the active learning technique. There is an enhancement of the threats detection results while a considerable decrease of the costs and amount of time when labeling vast datasets which is why it might be regarded as effective and efficient solution.
- The proofs of the results demonstrate how the proposed technique was implemented, showing that the Random Forest classifier, with an accuracy of 83.91%, is the best performer in detecting adversarial risks within AVP systems. This highlights the practical applicability of the outlined approach in enhancing the security and safety of AV systems.

This research work is organized into five main sections:

- The first section (Gupta et al., 2020) outlines the background, motivation and contribution of the study.
- The second section (Abramson, 2016) presents the related studies on adversarial attack detection in autonomous systems.
- The third section (Gupta et al., 2020) is the proposed approach where we explain the machine learning and active learning techniques that were used to detect the adversarial attacks.
- In the fourth section (Sun N. et al., 2023) on the experimental analysis and result, various classifiers' performances are compared.
- Last but not least, the final section offers a discussion on the major research implications before giving an overall conclusion in the fifth section (Anwar, 2023).

## 2 Literature review

Dadras et al. (2018) aim at designing a suitable method for identification of the attacker in the Cyber-Physical Systems (CPS). In more detail, the author presents a detection algorithm that utilizes system identification methodologies in conjunction with machine learning to foster security in CPS. The system model is linear time invariant, and the attacker has the capability to disturb the performance of the system through control input and gain values. To identify anomalous subsystems from a power system, the author proposes a new method of state- space and transfer function identification to compare various parameters of subsystems. In the method no prior information about the amount or the location of the attackers is needed, and the method is computationally efficient. The success of this approach is illustrated via the vehicular platooning in an adversarial context, where the algorithm for the identification of the platoon members, and for the detection of malicious actors is based on the analysis of the deviations of the eigenvalues, and other system characteristics. Finally, the paper concludes that the method is effective for identifying gain modification and destabilizing attacks providing a real solution for increasing CPS protection.

Aliwa et al. (2021) comprehensively survey the security challenges, with emphasis on Controller Area Network (CAN) Bus networks. CAN is an automotive bus standard that enables micro-controllers and devices to interact with each other without the need for a host computer. CAN is a message-based protocol, which means that the communication takes place via the transmission of messages that are being cast to all nodes in the bus rather than being relayed directly from one to another node. Each node in the bus assesses whether to receive the message depending on its identification. There are no inbuilt security factors, which also introduces possibilities for security exploits. As automobiles and the systems, they contain grow more linked via “different interfaces such as Bluetooth, Wi-Fi or OBD-2 interface,” they are more vulnerable to external attacks. Since CAN Bus is despite its lack of security factors the most used bus for communicating with vehicles, attackers can easily perform manipulative attacks like message injecting, creating a DoS condition, or penetrating CAN sniffing attacks. The author surveys currently known cryptographic methods as well as Intrusion Detection Systems IDSs intended to counteract these threats while comparing their real-time performance, hardware load, and efficiency. The survey divides them into physical and remote entry points for cyberattacks, targeting keyless fob attacks, GPS spoofing attack, and the tire pressure monitoring system (TPSM) attack. The paper also assesses how well current countermeasures like lightweight cryptography and IDS-based techniques fare and how the paper identifies their drawbacks and suggests future research focus for improving in-vehicle cybersecurity.

Müter et al.'s (2011) paper investigates an anomaly detection scheme for in-vehicle networks with concern to security in autonomous vehicle platooning. In the method, a monitoring system employing sensors installed in various vehicle systems monitors network traffic with sensor data is used to identify disturbances which may correspond to adversarial threats. The detection system is real-time measuring network anomalies without producing false alarms through comparing traffic with standard protocol and system behavior. In this paper, it is ascertained that the proposed system accurately recognizes threats to improve safety and security of vehicle against cyber threats in complex interconnected environment. The same authors (Müter et al., 2010) present a comprehensive framework for defining and detecting anomaly in in-vehicle networks, with a view to improving the security of what is today's advanced vehicles and related adaptive solutions. And it proposes a new set of sensors meant to capture different sorts of networking irregularities such as message forms, location, type, frequency and so on without the production of false alarms. Such sensors assist in determining whether attacks such as message injection or manipulation that would endanger vehicle safety are likely to occur. The approach is intended to establish real-time detection as part of automotive network security architectures to respond to cyber threats efficiently. Tomlinson et al. (2018) discuss the specific issues of using IDMs in the automotive CAN. This re-establishes various forms of the intrusion detection manner which are signature-based and the anomaly-based detection manners which include the statistical techniques, clustering and mechanical learning. These methods are assessed with regard to their feasibility for the implementation at the onboard vehicle ECUs in the field of constraint hardware resources. The challenges noted in the course of the research include the method of labeling attacks and the need for efficient updating and reliable detection of the attacks.

Sun et al. (2022) propose an approach to improve the CPS's security against adversarial attacks by using a mixture of methods. A deep learning (DL) together with the physical dynamics knowledge is targeted to enhance attack detection effectiveness and strengthen the security of the system. In simulating an autonomous vehicle platoon, the author shows the practical applicability of the proposed approach by highlighting the better F1 score that is twice the baseline and improved distances between vehicles in the platoon. This implies that the proposed hybrid approach can have a better and more effective defense mechanism in cases of CPS adversarial attack. In this paper (Sun G. et al., 2023), the authors introduce a new method to mitigating the security threats that can affect vehicle platooning systems.

Finally, based on the machine learning, control theory and game theory, the author proposes a complete solution for attack detection and avoidance. To represent the relationships between attackers and defenders, the work formulates and solves a non-cooperative security game with incomplete information, which supports intelligent decision-making on detector placement and attack prevention. Further, the paper presents a control system reconfiguration method to counter threats and offers a stability review. Results show that the proposed method achieves better inter-vehicle distance as well as defense utilities and is more robust against ambient traffic compared to baseline defenses. This implies that the game-based defense formulation can afford a more optimal and robust shield to the vehicle platooning systems from adversarial attacks.

Furthermore, more real-world Autonomous Vehicle Platooning (AVP) case studies could improve the practical foundation of adversarial threat detection methods. Recent deployments provide vital information on real-world difficulties and potential weaknesses. For example, the ENSEMBLE project (Cordis, 2024) extensively tested truck platooning across Europe, highlighting not only fuel efficiency and traffic improvements, but also the critical role of cybersecurity in platooning scenarios, particularly in terms of vehicle-to-vehicle (V2V) communication threats (Axelsson, 2017).

Similarly, recent autonomous truck platooning deployments in the United States also provide evidence of powerful adversarial threat detection systems that are real-time capable. For example, the most recent evaluations conducted under the US Department of Transportation's Automated Driving Systems (ADS) Demonstration Grants have documented several practical cybersecurity vulnerabilities, such as V2V communication interruptions, GPS spoofing, and sensor manipulation attempts in live operational environments (Garrett et al., 2023).

These studies highlight the significance of verifying theoretical threat models against real-world scenarios in order to assure the resilience and dependability of AVP systems (Table 1).

**Table 1:** Comparison of existing AVP intrusion-detection approaches with our proposed method.

**Reference/paper**	**Primary focus/problem addressed**	**Methodology/technique used**	**Application domain**	**Data requirements/labeling**	**Key strengths**	**Limitations/gaps**	**This work's contribution/improvement**
Dadras et al. (2018)	Attacker identification in Cyber-Physical Systems (CPS)	System Identification + Machine Learning (based on eigenvalue deviations and system characteristics). Detecting and localizing the attacker is done with K-means clustering and thresholding.	General CPS having data like states including velocity and position (particularly in vehicular platooning)	Input-output data of each subsystem for parameter identification, for vehicular platooning it includes vehicles states. No prior knowledge of the number of attackers or the system's normal or adversarial parameters required.	Novel thresholding/clustering for attack detection, Computationally efficient; effective for identifying gain modification and destabilizing attacks, relatively high detection rates compared to the standard State Space Identification.	General CPS focus, not exclusively AVP adversarial threats; potential need for labeled data for ML components.	Focuses specifically on AVP adversarial threats; enhances data efficiency via Active Learning. Ness' approach intelligently selects the most informative data samples for labeling, significantly reducing the manual effort and cost associated with building large, labeled datasets for machine learning models, a crucial practical advantage in AVP security where attack data can be scarce.
Aliwa et al. (2021)	Security challenges & countermeasures in (inherently vulnerable) Controller Area Network (CAN) Bus networks on real-time constraints, hardware used, changes in CAN Bus behavior, types of attack mitigation	Comprehensive Survey of Cryptographic Methods & Intrusion Detection Systems (IDSs) based on anomaly detection (statistical, machine learning, rule based, physical fingerprint methods, signature-based detection)	Automotive CAN Bus, extendability to other serial protocols like LIN and FlexRay and built environments, railway applications, medical devices, aircrafts.	Survey. Statistical IDS uses CAN ID Frame/Frame Floe behaviors. Machine Leaning IDS uses labeled and raw CAN Data, supervised ML require labeled attack data. Physical IDS requires physical statistical features like skew, clock offset, and clock frequency.	Comprehensive overview of CAN security; compares real-time performance, hardware load, and efficiency of countermeasures.	Survey, not a novel detection framework; primarily focuses on CAN physical layer vulnerabilities. Hardware-based cryptography, while potentially faster, is not compatible with current vehicles due to the need for ECU updates, and the cost of implementation can be significant.	Provides a novel, integrated framework for AVP, addressing higher-level communication threats and broader adversarial attacks beyond CAN that allows only low bandwidth, small frame size and limited computational resources, with high real-time sensitivity. Aliwa et al. notes that hardware-based cryptographic solutions are often incompatible with existing ECUs and costly. This Work's ML-based approach is more flexible and software-centric and avoids such hardware update issues. It also explicitly mitigates the “time-consuming” nature of labeling raw CAN data for supervised ML, which Aliwa et al. highlights, through its Active Learning component.
Müter et al. (2011)	Anomaly detection scheme for in-vehicle networks; comprehensive framework for defining and detecting anomalies using a reactive approach.	Monitoring system with sensors; compares network traffic with standard protocol/system behavior; new sensors for network irregularities (protocol specifications, sub-network or domain, data type in the message payload, data range, frequency, correlation, traffic order and timing, correlations with previous values, redundant/duplicate information from vehicle data)	In-vehicle networks (with concern for AVP security)	Assumes knowledge of standard protocol/system behavior for comparison. For each sensor specific data is required for detection.	Real-time network anomaly detection; high accuracy without false alarms if comparison data is correct; effectively recognizes threats; assists in detecting message injection/mani-pulation.	Primarily focused on general in-vehicle network anomalies; not explicitly designed for specific adversarial attack types in AVP context, nor data labeling efficiency through active learning. Difficulty distinguishing attacks from hardware errors. Difficulty of physical layer monitoring due to frequent changes in signal characteristics from environmental variations.	Specifically targets adversarial threats in AVP using active learning for data efficiency. Can detect anomalies where Müter et al.'s method fails due to lack of historical and comparison data. More robust against dynamic threats.
Tomlinson et al. (2018)	Specific issues of using Intrusion Detection Methods (IDMs) in automotive CAN.	Survey methodology. Discussion of signature-based and anomaly-based detection (statistical, clustering, ML). Assessment of feasibility for onboard vehicle ECUs.	Automotive CAN Bus	Implicitly relies on labeling attacks for signature/anomaly detection. This data includes network traffic data, expected behavior profiles, labeled attack data and contextual information that may be difficult to obtain.	Comprehensive discussion of IDMs; assesses feasibility for ECU implementation despite hardware constraints.	Challenges noted in labeling attacks; need for efficient updating and reliable detection; difficulty in obtaining and generating attack data; focuses on CAN layer, not higher-level AVP communication protocols.	Offers a novel, data-efficient framework specifically for AVP threat detection using active learning. Addresses the identified gaps by incorporating Active Learning, which significantly reduces the dependency on large, pre-labeled datasets and streamlines the data acquisition and labeling process for machine learning models, making the approach more practical. This work extends its focus to higher-level AVP communication protocols (V2V, V2I), which are crucial for platooning coordination and represent a more complex attack surface that involves more than just low-level CAN frames.
Sun G. et al. (2023)	Improve CPS security against adversarial attacks; enhance attack detection effectiveness.	Deep Learning (DL) + Physical Dynamics Knowledge, including hybrid attack detection, physical dynamics layer, deep learning model, adversarial training and an attack model. Testing using a simulator for an autonomous vehicle platoon test-bed.	Autonomous Vehicle Platooning (AVP)	Implies need for data for DL training, including clean data samples, adversarial examples, physical system parameters, input-output data.	Hybrid approach combining physics and control theory with deep learning; improved inter-vehicle distances; practical applicability in AVP simulation.	Potential high data dependency for DL; specific attack types may not generalize; complexity of integration; can be circumvented with new attack vectors, may lack active learning benefits.	Our active learning approach significantly reduces the need for large, labeled datasets. Better adaptability to Novel Attack Vectors.
Sun N. et al. (2023)	Mitigating security threats affecting CPS, i.e., vehicle platooning systems.	New method combining system identification methods with machine learning techniques. Data collection, parameter identification, comparison of parameters or eigenvalues and subsequently anomaly detection.	Vehicle Platooning	Input-output data of each subsystem for parameter identification. The paper uses 1,000 data sets of a 101-vehicle platoon generated via Monte Carlo simulation for various attacker positions and numbers.	Proposes a new method for mitigating threats. Versatility against both destabilizing and gain modification attacks.	Generalizabilty of parameters, chouce of parameters for detection might be application-dependent and requires careful selection, Assumption of Attacker Minority, “Trial and Error” for Coefficient k, assumes known system order.	Provides a concrete framework with empirical results and specific ML classifiers for threat detection. his approach can reduce integration complexity and computational overhead for real-time detection, especially when the primary goal is robust attack detection rather than complex game-theoretic mitigation strategies that might require extensive real-time computation.
This Work (Ness et al.)	Robust Detection of Adversarial Threats in Autonomous Vehicle Platooning	Active Learning + Ensemble Machine Learning Classifiers (RF, GB, XGB, KNN, LR, AdaBoost)	Autonomous Vehicle Platooning (AVP)	Significantly reduced requirement for labeled data via Active Learning	Achieves high detection accuracy (83.91% with RF); addresses data labeling bottleneck; comprehensive multi-classifier evaluation; handles uncertainty efficiently.	Current focus on algorithmic efficacy; real-time computational overhead for deployment is a future work.	N/A

## 3 Proposed approach

Innovative and unique to the approach discussed is the integration of active learning with machine learning classifiers. The active learning component of the model actively requests the labels of the most informative or less confidently classified data points in the detection process, thus making it much more efficient. This approach cuts down the amount of labeled data required and makes it possible for the classifiers to reserve their efforts for important complex adversarial cases.

Therefore, the integration improves the speed and performance of identification of threats, increases the rate of yields, and reduces labeling expenses, effortlessly making more effective models.

Only labeled data is used to train the classifier in a machine learning approach called uncertainty sampling. The approach then predicts probabilities on test data and calculates uncertainty by selecting the sample with the highest prediction probability and lowest model confidence.

The model learns better on these difficult conditions by training the examples with the most confusing sample and excluding it from testing. The best sequence is chosen after measuring model performance using accuracy, F1, precision, and over-recall at the end of each iteration. Thus, each integration speeds up threat identification, boosts yield, lowers labeling costs, and creates more effective models easily.

To clarify, Algorithm 1 shows the approach.

Algorithm 1 Active learning with machine learning classifier for multi-class classification.

**Study**   **Algorithm used**
1. *Input*:*feature set X, target labels y*, *number of iterations n*
2. *Initialize lists*:*accuracies, f*1 < *uscore* > *measure* < *uscore* > *list, precision* < *uscore* > *list, recall* < *uscore* > *list*
3. *for i* = 1 *to n do*
4. (*X*_*train*_, *X*_*test*_, *y*_*train*_, *y*_*test*_)←*rain*_*test*_*split*(*X, y*)
5. *Reset indices for X*_*train*_, *X*_*test*_, *y*_*train*_, *y*_*test*_
6. *Initialize classifier model*
7. *model*.*fit*(*X*_*train*_, *y*_*train*_)
8. *p*←*model*.*predict* < *uscore* > *proba*(*X*_*test*_)
9. Compute uncertainty as max probability: *u*←max(*p*)
10. Identify the most uncertain sample: *s*_*uncertain*_←argmin(*u*)
11. Add uncertain sample to training data:
12. *X*_*train*_←*X*_*train*_∪*X*_*test*_[*s*_*uncertain*_]
13. *y*_*train*_←*y*_*train*_∪*y*_*test*_[*s*_*uncertain*_]
14. Remove uncertain sample from test set:
15. *X*_*test*_←*X*_*test*_\*X*_*test*_[*s*_*uncertain*_]
16. *y*_*test*_←*y*_*test*_\*y*_*test*_[*s*_*uncertain*_]
17. Predict on *X*_*test*_: ŷ←*model*.*predict*(*X*_*test*_)
18. *Compute accuracy, F*_1_, *precision, recall*
19. Append metrics to respective lists
20. end for
21. Identify best iteration based on highest accuracy i^*^←argmax(*accuracies*)
22. Output: Best accuracy and corresponding iteration *Output*:best accuracy accuracy^*^ = *accuracies*[i^*^], iteration i^*^

### 3.1 Dataset

The dataset used in Detect Adversarial Threats in Autonomous Vehicle Platooning is made available on Kaggle to allow researchers and practitioners to use it (Vaccari et al., 2022). It is organized into folders for different application scenarios, specifically platooning, and contains two main subfolders: one for legitimate data which determines non-tampered data, and the other for adversarial data, which signifies the data that was attacked by the adversarial machine learning. The dataset is expected to be analyzed methodically and for this purpose contains the training dataset, the testing dataset and the combined formatted dataset. Obviously, other features are incorporated in the simulation concerning the application of the platooning, for example, the number of cars in the platoon, the braking force, the packet error rate, as well as distance and speed parameters between the vehicles. This structured dataset has a noteworthy function for improving the identification of the adversarial threats in self-driving car convoys.

### 3.2 Data preprocessing

**Figure 1:**
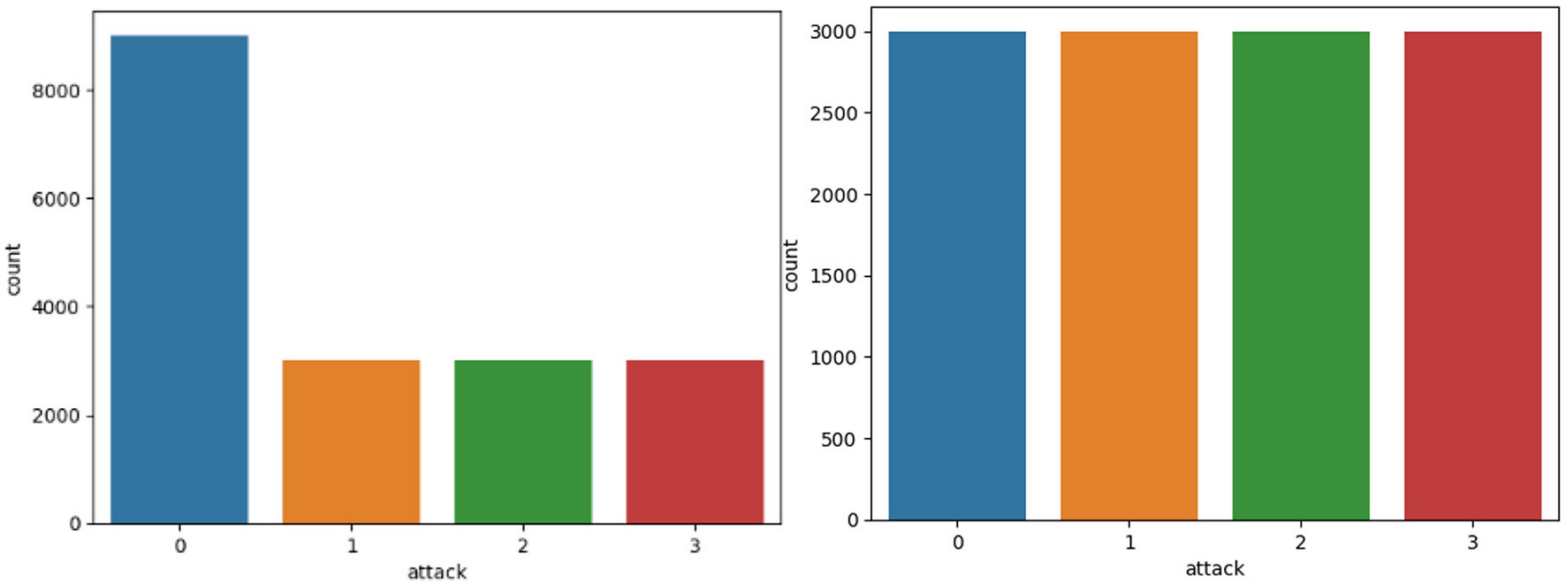
Distribution of attack class before and after data balancing.

**Figure 2:**
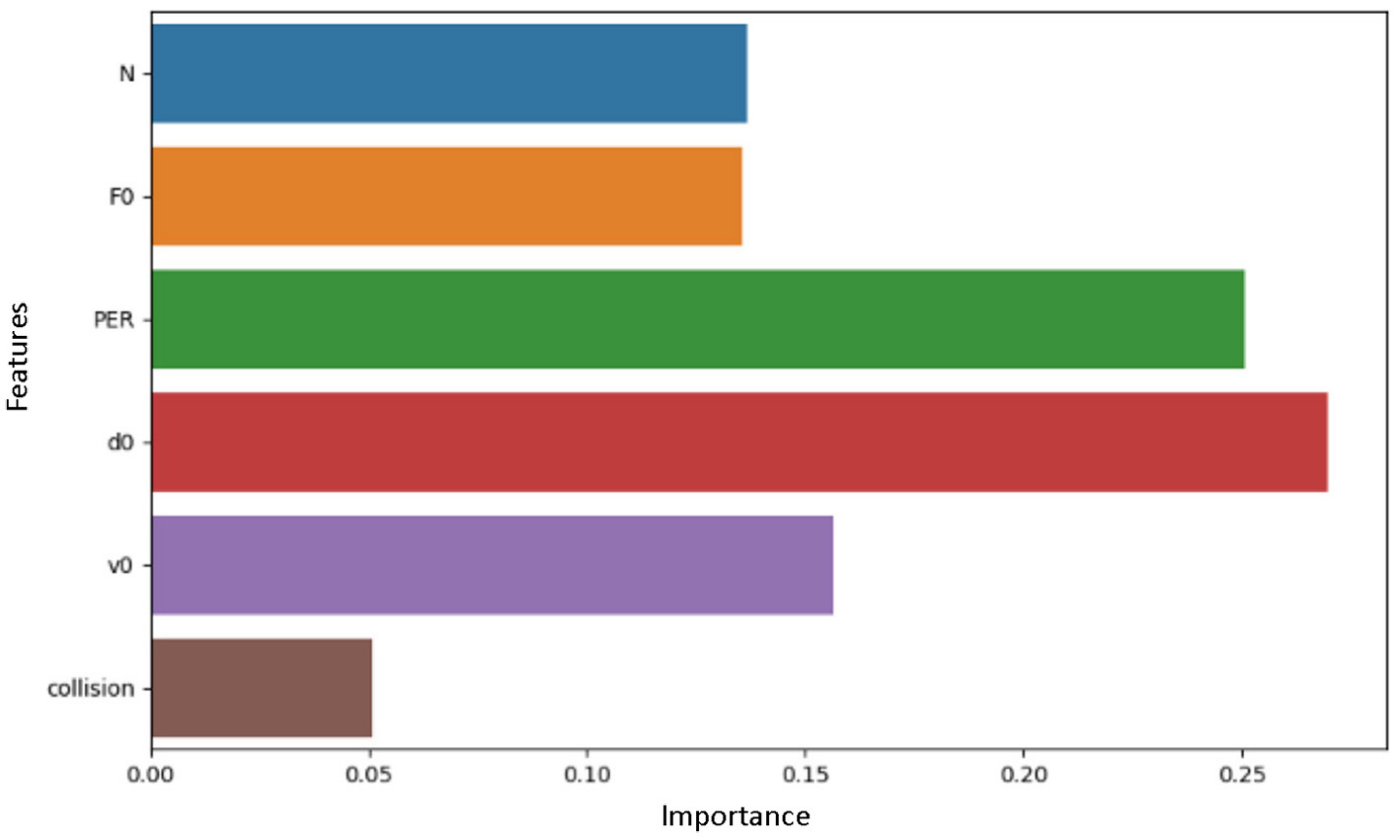
Selected features.

To optimize the performance as well as the steadiness of the said dataset, previous preparatory steps were taken. To reduce bias in this model, the rows were duplicated, then only one of them would be retained. Some of the pre-processing techniques implemented was to clean the data to eliminate any discrepancies or mistake in the features contained in a data set. The issue of missing and null values was also solved in this paper either by imputation or by deletion because such values may undermine the model outcomes, and the classifiers used must be provided with high quality input data. All these preprocessing steps help improve the robustness and accuracy of the machine learning model (see Figures 1 and 2).

### 3.3 Active learning

In active learning, the machine learning algorithm is hard coded in a way that it can ask a user or an oracle to label new points or examples (Ren et al., 2021). In contrast to the acquisition of a huge set of entirely labeled data, which can be time- consuming and costly, active learning chooses the most informative samples to label to learn the model better. It requires fewer labels to accomplish this goal. This methodology is most effective in playing multiple scenarios when labeled samples are difficult to come by or can be obtained only at a significantly higher cost. It also enables a faster tuning of the model when considering learning in certain instances to be uncertain or even completely difficult.

#### 3.3.1 RF with active learning

Random Forest has been modified with active learning to offer a powerful combination of ensemble learning techniques that aims to try out its best performance with small amounts of labeled data added to active learning. Random Forest combines a number of decision trees, each of which are built from a random sample of the training data and are combined to form the final model (Shaik and Srinivasan, 2019). When used together with active learning, the model is proactive in identifying samples that are informative or of high uncertainty which the Random Forest can then use to train on the most complex or least represented samples. This combination is especially useful for minimizing the costs of labeling while simultaneously creating an efficacious and resilient approximation model. Random Forest builds multiple decision trees and outputs the average prediction of the individual trees (for regression) or the mode of the predictions (for classification) as shown in Equation 1.


$$\hat{y}=\frac{1}{N}\sum_{i=1}^{N}T_{i}\left(x\right)$$ (1)

#### 3.3.2 GB with active learning

Gradient Boosting with active learning integrates the basic concept of GB into active learning to improve the gradient boosting model using as many labeled samples as possible. Like all boosting algorithms, Gradient Boosting creates a number of weak learners and then combines them to form a powerful model; the weak learners are usually decision trees (Bahad and Saxena, 2020). In its basic form, the model maps the examples. When combined with active learning, the most ignorable or most informative samples are used for labeling so that the training process is concentrated on the most difficult samples. This type of labeling is more targeted, which means that fewer labeled data are needed while achieving high predictive accuracy for the model. Gradient Boosting minimizes the loss function L by adding weak learners sequentially; the model updating is shown in Equation 2.


$$\begin{array}{l} F_{m\;}\left(x\right)=F_{m-1\;}\left(x\right)+\eta h_{m}\left(x\right) \end{array}$$ (2)

#### 3.3.3 XGB with active learning

XG-Boost, or Extreme Gradient Boosting, is a high- performance and scalable tool that is built completely from scratch for Gradient Boosting. When active learning is combined with XG-Boost, the model proceeds iteratively to concentrate on the data points, which may be the most difficult or which the model is least sure about (Margraf et al., 2024). This fine-tuning selection guarantees that the model refines its mistakes quickly; hence, fast convergence and precise results are achieved with fewer labeled data. Equation 3 shows that XG-Boost improves gradient boosting by introducing regularization to control overfitting.


$$\begin{array}{l} F\left(x\right)=\overset{M}{\underset{m=1}{\sum }}\gamma_{m}h_{m}\left(x\right) \end{array}$$ (3)

#### 3.3.4 KNN with active learning

The easiest and most versatile of all algorithms is the K- Nearest Neighbors (KNN), which classifies new information based on the closest labeled maps in the feature space (Boateng et al., 2020). In the active learning framework, KNN gains from querying the least certain or most ambiguous examples for their labels. This helps them make their boundary regions more accurate and improve their classification than if they have to rely on a large number of labeled data. KNN predicts the class by considering the majority class among the k-nearest neighbors, shown in Equation 4.


$$\begin{array}{l} \hat{y}=arg\;max_{c}\underset{i\epsilon N_{k}\left(x\right)}{\sum }I\left(y_{i}=c\right) \end{array}$$ (4)

#### 3.3.5 LR with active learning

Logistic Regression (LR) is a simple linear model that is often used in situations where the output is a probability estimation of the classes (Boateng and Abaye, 2019). When applied with active learning, Logistic Regression identifies the data points closer to the decision boundary, where the model is most uncertain. This targeted labeling enhances the model performance with less labeled data because the decision boundary is finely tuned precisely where it is needed most. Logistic Regression predicts the probability of a binary outcome using the logistic function that can be seen in Equation 5.


$$\begin{array}{l} P\left(y=1|x\right)=\frac{1}{1+e^{-\left(\beta_{0}+\beta_{1}x_{1}+\cdots +\beta_{n}x_{n}\right)}} \end{array}$$ (5)

#### 3.3.6 AdaBoost with active learning

AdaBoost (Adaptive Boosting) is one of the most powerful ensemble learning methods where weak learners, including the decision trees, are applied and enhanced according to the improvement of weighted accuracy (Mienye and Sun, 2022). In active learning, AdaBoost asks selections of the most difficult or most uncertain samples to label so that each subsequent weak learner learns most, where the model is weak. Thus, we also examine how this adaptive process along with selective labeling yields a very accurate model with few labeled instances. Equation 6 shows AdaBoost combining weak learners iteratively by adjusting their weights based on the errors of previous learners.


$$\begin{array}{l} F\left(x\right)=\overset{M}{\underset{m=1}{\sum }}\alpha_{m}\gamma_{m}h_{m}\left(x\right),\;\alpha_{m}=\frac{1}{2}\ln\left(\frac{1-\epsilon_{m}}{\epsilon_{m}}\right) \end{array}$$ (6)

## 4 Experimental analysis and results

In the experimental part of the paper “A Structured Approach to Detect Adversarial Threats in Autonomous Vehicle Platooning,” many classifiers were used such as XG-Boost, K- Nearest Neighbors (KNN), Logistic Regression (LR), and AdaBoost all using active learning. It was observed that different models yielded diverse levels of performance—accuracy and operation efficiency. The incorporation of active learning throughout all classifiers helped gain large improvements in threat detection capabilities by minimizing the amount of training data required while simultaneously boosting predictive efficiency. In general, the models proved their ability to identify adversarial threats and illustrated inactionable potential of active learning for enhancing the efficiency of various machine learning techniques.

### 4.1 Performance matrix

Outcomes are important for assessing the performance of machine learning classifiers, as they measure a model's ability to predict. The above are useful in evaluating a classifier's performance on a given dataset, as well as making decisions about classifier selection, optimization, or modification. General performance indicators include model accuracy, precision, recall, and the F1 value, each measuring a different aspect of the model's performance. By measuring these indicators, we can determine how well a classifier will perform on unseen data, its ability to identify specific classes, and the trade-off between false positives and false negatives.

Accuracy is calculated by dividing the overall rate of correct classification by the number of true positive and true negative figures in the model's positive and negative classifications, respectively. Precision focuses on the ratio of correct positive predictions, which provides the percentage of true positives out of all positives predicted by the model.

Recall, also known as sensitivity, measures the model's ability to avoid overlooking true positives and provides the percentage ratio of true positive results to the actual total number of positive results. The F1 score is the mean of precision and recall when both values are useful and there is an imbalance between classes.


$$\begin{array}{r} \text{Accuracy}=\frac{TP}{TP+TN+FP+FN}\\ \text{Precision}=\frac{TP}{TP+FP}\\ Recall=\;\frac{TP}{TP+FN}\\ F1-Score=\;\frac{2\;\cdot \left(Precision.Recall\right)}{Precision+Recall} \end{array}$$

A confusion matrix is a table that provides a detailed analysis of classifier results in terms of true positives, false positives, and false negatives. It aids in representing the distribution of prediction errors and identifying areas where the model is ineffective.

The ROC curve, also known as the Receiver Operating Characteristic curve, compares the true positive rate (recall) to quantities equal to the false positive rate.

The area under the receiver operating characteristic curve (ROC) is used to assess the model's overall predictive accuracy without specifying a threshold; the larger the area, the better the model's ability to distinguish between the positive and negative classes.

### 4.2 Results

**Figure 3:**
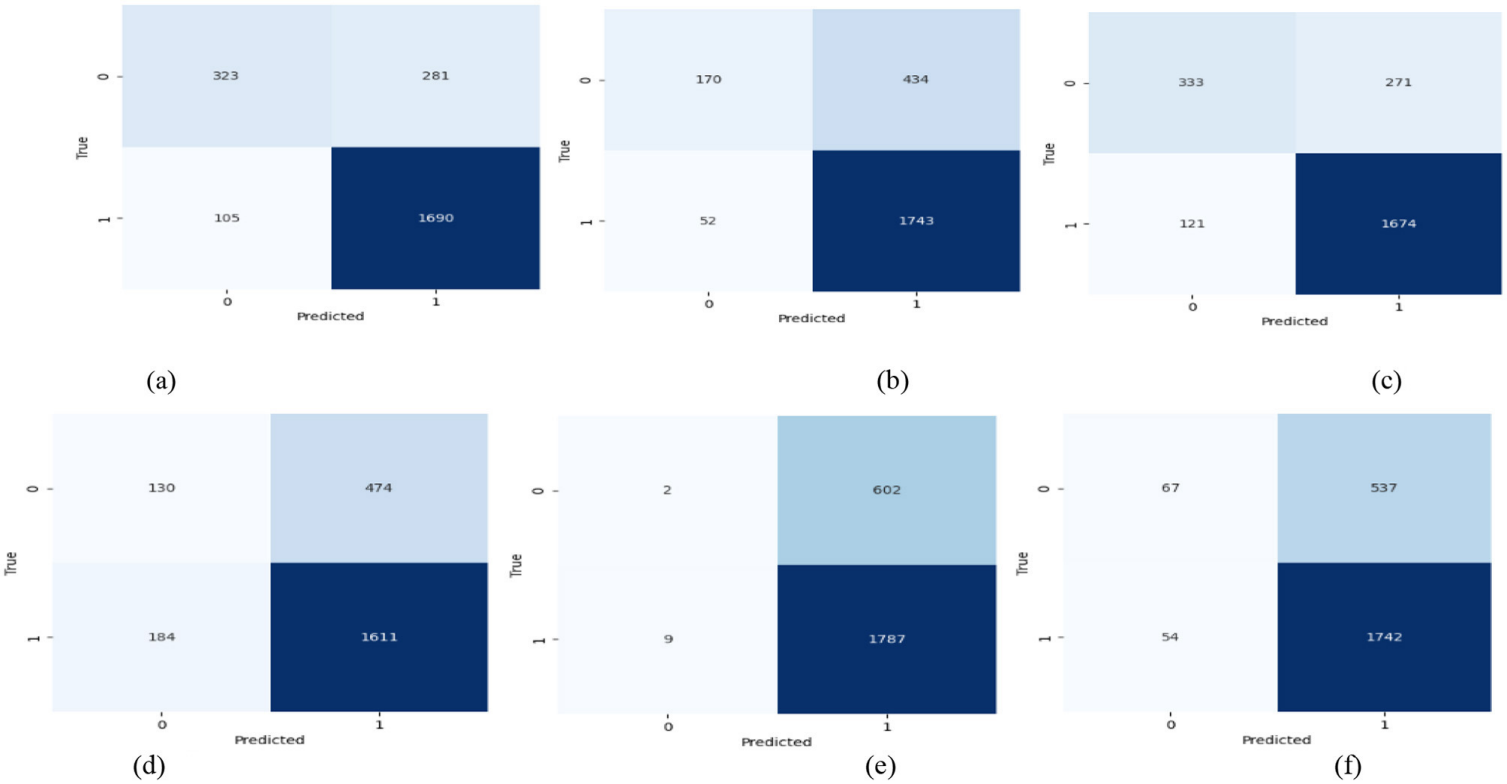
Confusion matrix of active learning with ML classifiers. **(a)** RF, **(b)** GB, **(c)** XGB, **(d)** KNN, **(e)** LR, **(f)** AdaBoost.

The number of features in the following classifiers for detecting adversarial threats in autonomous vehicle platooning demonstrate that the tested models are used to classify the data. This takes the form of a confusion matrix, in which some models scored high in correct positive prediction but low in false negative. As shown in Figure 3, RF and XGB are expected to produce confusion matrices that are nearly balanced, with the greatest number of correctly classified samples and the fewest incorrectly classified. Other classifiers, such as K-Nearest Neighbors (KNN), may produce high imbalanced confusion matrices with misclassification tendencies and high levels of errors, especially when operating on complex or ambiguous adversarial threats.

In Figure 3, each confusion matrix displays the classification performance of the following classifiers: (a) Random Forest (RF), (b) Gradient Boosting (GB), (c) Extreme Gradient Boosting (XGB), (d) K-Nearest Neighbors (KNN), (e) Logistic Regression (LR), and (f) AdaBoost. The x-axis represents predicted labels, while the y-axis represents true labels, showing correct as well as incorrect classifications. These matrices show each classifier's accuracy and misclassification rates in detecting adversarial threats in AVP systems.

**Figure 4:**
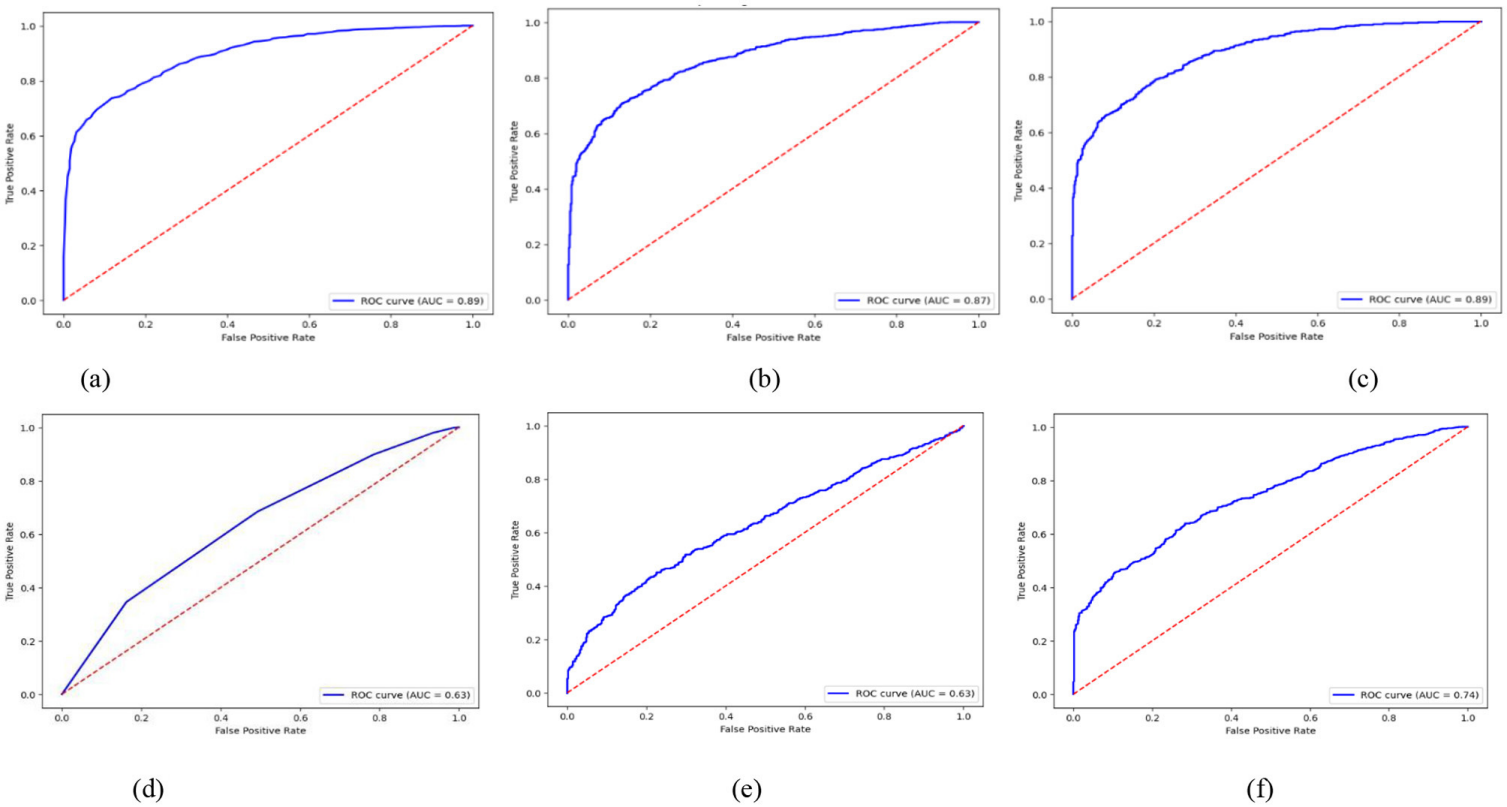
ROC curves of active learning with ML classifiers. **(a)** RF, **(b)** GB, **(c)** XGB, **(d)** KNN, **(e)** LR, **(f)** AdaBoost.

It is expected that RF and XGB models will have high ROC curves and few false positives when distinguishing adversarial from non-adversarial instances. These models must have a larger Area under the curve (AUC), which is expected given their ability to maintain consistent performance across a range of decision thresholds, as illustrated in Figure 4. Instead, some models, such as KNN and Logistic Regression (LR), may produce relatively low ROC curves, indicating that the classifiers perform poorly for class separation, particularly under difficult detection conditions.

In Figure 4, the ROC curves depict the true positive rate (sensitivity) against the false positive rate (1-specificity) across various threshold settings for classifiers: (a) Random Forest (RF), (b) Gradient Boosting (GB), (c) Extreme Gradient Boosting (XGB), (d) K-Nearest Neighbors (KNN), (e) Logistic Regression (LR), and (f) Adaptive Boosting (AdaBoost). Each curve has 95% confidence interval (CI) error bands around the Area Under the Curve (AUC). This shows that the classifier is strong and can tell the difference between threats and non-threats in AVP settings.

The explanation of these models in terms of ROC curves showed that under adversarial threat modeling, classifiers must possess significant discriminatory thresholds for the platooning of autonomous vehicles.

The precision score of the Random Forest (RF) classifier for class 0, non-attack data, in this case, was 75%, while for class 1, attack data, we see that the precision score was 86% as shown in the Table 2. From this, we are able to conclude that the RF model has a tolerable performance in detecting attack instances, though, at the same time, it is fairly efficient in identifying non-attack data. The balance of precision values means that RF is able to generalize well across both classes and is, therefore, very valuable for AV security applications. Also, its ensemble learning capability appears to enable it to work with numerous decision trees and increase its resistance to adversarial risks. Specifically, these precision scores indicate that RF has the potential to greatly contribute toward the improvement of the safety and security of autonomous vehicle platooning systems through successful discrimination between normal and anomalous behaviors.

**Table 2:** Classification report of random forest.

**Class**	**Precision**	**Recall**	**F1-score**	**Support**
0	0.75	0.53	0.63	604
1	0.86	0.94	0.90	1,795
Macro avg	0.81	0.74	0.76	2,399
Weighted avg	0.83	0.84	0.83	2,399

The Gradient Boosting (GB) classifier shows a precise value of 77% for class 0 and 80% for class 1, clearly reflecting that it is very effective in detecting both normal flow (non- attack) and the attack as shown in the Table 3. This dual utility is very useful for applications that require identification be- tween normal and adversarial activities. Due to the sequential learning technique, it becomes easy for the GB to learn from misclassified instances in order to improve its ability to make better predictions with subsequent iterations. A slightly better accuracy for the attack data indicates that GB could benefit from PGM in identifying threats concerning self-driven cars, especially in terms of fast detection of hostile actions, which could reduce the dangers involved. Moreover, the balanced precision values stress the accuracy of GB, which might help this technology become a useful instrument in the context of cybersecurity for the protection of the AV systems for platooning against new threats.

**Table 3:** Classification report of gradient boosting.

**Class**	**Precision**	**Recall**	**F1-score**	**Support**
0	0.77	0.28	0.41	604
1	0.80	0.97	0.88	1,795
Macro avg	0.78	0.63	0.64	2,399
Weighted avg	0.79	0.80	0.76	2,399

In the Extreme Gradient Boosting (XGB) classifier the precision for the class 0 was 73% and for the class 1 it was as high as 86% (Table 4). This suggests that although XGB performs very well in recognizing instances of attack, there is a reasonable tradeoff in recognizing non-attack data. By using gradient boosting framework, XGB can easily reduce the loss functions resulting into high performance especially when identifying adversarial threats. The strong precision for attack data represents the ability to find and prevent malicious actions, which is essential for the security of AVP. However, it has rather slightly lower precision for non-attack instances, what point at the necessity of further improvement of the method in the classification of abnormal actions and, therefore, emphasizes the necessity of continuous model improvement in conditions of dynamic capabilities.

**Table 4:** Classification report of extreme gradient boosting.

**Class**	**Precision**	**Recall**	**F1-score**	**Support**
0	0.73	0.55	0.63	604
1	0.86	0.93	0.90	1,795
Macro avg	0.80	0.74	0.76	2,399
Weighted avg	0.83	0.84	0.83	2,399

The K-Nearest Neighbors (KNN) classifier shows a fairly lower precision of 41% for class 0 and 77% for class 1 as described in Table 5. The above results show that when it comes to non-attack cases, KNN often makes wrong predictions, which leads to the question of the practical applicability of KNN in environments where the differentiation between intermediate and attack behaviors is critical. The drawback is that KNN can be sensitive to the curse of dimensionality as experienced when working on high-dimensional data sets common with a cyber security application. As it can reasonably well-recognize attack instances, it has a problem with the non-attack data, which cause high false positive rates and, therefore, could increase the number of alerts and even system downtimes. Therefore, KNN can be used as an additional tool; however, due to low precision, its application in the autonomous vehicle platoon requires adequate consideration.

**Table 5:** Classification report of k-nearest neighbor.

**Class**	**Precision**	**Recall**	**F1-score**	**Support**
0	0.73	0.55	0.63	604
1	0.86	0.93	0.90	1,795
Macro avg	0.80	0.74	0.76	2,399
Weighted avg	0.83	0.84	0.83	2,399

As currently constructed, Logistic Regression (LR) has a notably low precision of 18% of classifying instances as class 0—which clearly presents difficulties in well-categorizing non-attack instances. On the other hand, there is a fair degree of accomplishment regarding attack data identification that is moderated by a precision of 75% for class 1 as described in Table 6. Different levels of precision, which are obtained when applying the algorithm, might be worrisome due to the fact that LR could not be effective in real-life cybersecurity use cases since data in these contexts might be considerably different from the data employed in the context of this study. It might be probable that analyzing certain data distribution through a linear approach may not be very effective since it fails to generalize the results studied through this model, as is observed in the case of LR. Therefore, even though continued usage of LR can augment the ability to recognize simplistic relationships existing within the data, it is not as precise as required for using it as the sole classifier of adversarial threats in autonomous vehicle platooning systems.

**Table 6:** Classification report of logistic regression.

**Class**	**Precision**	**Recall**	**F1-score**	**Support**
0	0.18	0.00	0.01	604
1	0.75	0.99	0.85	1,796
Macro avg	0.46	0.50	0.43	2,400
Weighted avg	0.61	0.75	0.64	2,400

AdaBoost classifier yields a precision of 55% for class 0 and 76% for class 1, which shows that it can analyze and recognize attack instances but struggles in differentiating between non-attack data and instances as shown in the Table 7. This difference in sharpness is in harmony with the model's capabilities: it is good at recognizing threats while lacking the ability to decipher benign cases. The reason AdaBoost is an ensemble learning algorithm makes it more robust by forming a strong predictor from multiple weak learners. However, as was seen, the average precision for anything but an attack instance is considerably lower, which brings into question its applicability in cases where fine distinctions between normal and malicious behavior are necessary. Therefore, the results imply continuous model assessment and recalibrations since AdaBoost should be flexible to accommodate new adversarial threats in the autopilot formation of self-driving cars and reduce fake alerts.

**Table 7:** Classification report of AdaBoost.

**Class**	**Precision**	**Recall**	**F1-score**	**Support**
0	0.55	0.11	0.18	604
1	0.76	0.97	0.85	1,796
Macro avg	0.66	0.54	0.52	2,400
Weighted avg	0.71	0.75	0.69	2,400

### 4.3 Discussion

In the accuracy comparison of machine learning classifiers with active learning to Detect Adversarial Threats in Autonomous Vehicle Platooning, the Random Forest (RF) with active learning achieved the highest accuracy at 83.91% as shown in the Table 8. Overall, based on the number of iterations needed for convergence, the proposed approach demonstrates its enhanced ability to identify adversarial threats. In second place is XG-Boost (XGB), with active learning having a classification accuracy of 83.66%. Gesture Recognition using Gradient Boosting (GB) with active learning comes second, though slightly lower at 79.74%. The following other models: K- Nearest Neighbors, Logistic Regression, Ada-Boost and active learning give comparatively lower ac- curacy: KNN = 72.57%, Logistic Regression = 74.54%, Ada Boost = 75.38%. In general, it is noted that RF and XGB with active learning are the most effective, which means that it is best suited for enhancing threat identification for AV platooning (Tables 1, 4, 8).

**Table 8:** Accuracy comparison of ml classifiers with active learning.

**Classifiers**	**Accuracies**
RF with AL	83.91%
GB with AL	79.74%
XGB with AL	83.66%
KNN with AL	72.57%
LR with AL	74.54%
AdaBoost with AL	75.38%

## 5 Conclusion and future scope

This paper provides a systematically developed and highly efficient method of detecting adversarial threats in the emergent technology known as Autonomous Vehicle Platooning (AVP). Since communication in AVP systems is based on V2V and V2I, these systems are exposed to adversarial attacks that can affect the availability, integrity, and confidentiality of communicated data. These attacks when successful lead to undesirable consequences, such as car accidents, traffic jams or even complete system breakdown and loss of confidence in the autonomous driving technologies. This research therefore employs active learning with six classifiers, including Random Forest, Gradient Boosting, XG- Boost, K-NN, Logistic Regression and AdaBoost, to improve the detection performance of such adversarial threats. Active learning therefore assists the system in picking out the most relevant samples, and therefore it is not necessary to have a lot of labeled samples in order to achieve good classifiers. Cross validation of all the tested models pointed toward the Random Forest with active learning as the superior model with accuracy of 83.91% and is thus a feasible solution for accurate real-time threat detection in AVP systems.

These experiments prove that this method can effectively distinguish the normal and malicious behavior in AVP and improve the general safety and reliability of autonomous vehicle platoons. The effectiveness of this approach not only helps to solve the existing problem of security in AVP systems but also present a versatile structure that can be used in other fields of autonomous systems and cyber-physical security. The use of such a detection mechanism is thus essential for making protocols enabling the integration of AVP systems to contemporary traffic conditions resistant to adversarial control. Yet, the studies point out that achieving promising results, the protection of AVP Systems from a diverse array of complex attacks is far from trivial. Even though, the proposed method demonstrates superior performance, within the constrained environment of simple car- following scenarios, the real-world driving environment is much more complex and consequently the performance of the proposed method in real high-stake scenario remains uncertain and deserves more research. Besides this, since the adversarial tactics are dynamic with their operations, there is equal need to develop what can be referred to as dynamic defense mechanisms and hence do more research on such flexible security solutions.

Based on the proposed methodology, several future directions and development are outlined as follows. First, extending the database to encompass a variety of more intricate and varied attack types might increase the efficacy of the threat identification models. Second, more realistic pilots of this system that incorporate real-world dynamic and large-scale AVP applications must be conducted. Third, there is also a possibility to improve resistance to new generation adversarial approaches by incorporating hybrid machine learning methodologies including adversarial training. Fourth, future research could focus on optimizing computational overhead to ensure real-time responsiveness using hardware-software co-design solutions—such as dedicated accelerators or edge-computing architectures—, as this would further support active learning applications in real-time AVP scenarios. Finally, we propose the study of cross-layer security solutions that imply both physical and cybersecurity of the AVP systems, which will create the foundation for safer and more efficient fully autonomous transportation networks.

## Data Availability

The original contributions presented in the study are included in the article/supplementary material, further inquiries can be directed to the corresponding author.
